# Epigenetic regulation in myocardial infarction: Non-coding RNAs and exosomal non-coding RNAs

**DOI:** 10.3389/fcvm.2022.1014961

**Published:** 2022-11-10

**Authors:** Sara Fadaei, Fatemeh Zarepour, Mehrnoosh Parvaresh, Alireza Motamedzadeh, Seyed Saeed Tamehri Zadeh, Amirhossein Sheida, Mohammad Shabani, Michael R. Hamblin, Mehdi Rezaee, Maryam Zarei, Hamed Mirzaei

**Affiliations:** ^1^Department of Internal Medicine and Endocrinology, Shohadae Tajrish Hospital, Shahid Beheshti University of Medical Sciences, Tehran, Iran; ^2^Student Research Committee, Kashan University of Medical Sciences, Kashan, Iran; ^3^School of Medicine, Kashan University of Medical Sciences, Kashan, Iran; ^4^Department of Physical Medicine and Rehabilitation, School of Medicine, Isfahan University of Medical Science, Isfahan, Iran; ^5^Department of Internal Medicine, Faculty of Medicine, Kashan University of Medical Sciences, Kashan, Iran; ^6^School of Medicine, Tehran University of Medical Sciences, Tehran, Iran; ^7^Department of Anesthesiology, School of Allied Medical Sciences, Kashan University of Medical Sciences, Kashan, Iran; ^8^Laser Research Centre, Faculty of Health Science, University of Johannesburg, Doornfontein, South Africa; ^9^Department of Anesthesiology, School of Medicine, Shahid Madani Hospital, Alborz University of Medical Sciences, Karaj, Iran; ^10^Tehran Heart Center, Tehran University of Medical Sciences (TUMS), Tehran, Iran; ^11^Research Center for Biochemistry and Nutrition in Metabolic Diseases, Institute for Basic Sciences, Kashan University of Medical Sciences, Kashan, Iran

**Keywords:** myocardial infarction, non-coding RNAs, microRNAs, long non-coding RNAs, circular RNAs, exosome

## Abstract

Myocardial infarction (MI) is one of the leading causes of deaths globally. The early diagnosis of MI lowers the rate of subsequent complications and maximizes the benefits of cardiovascular interventions. Many efforts have been made to explore new therapeutic targets for MI, and the therapeutic potential of non-coding RNAs (ncRNAs) is one good example. NcRNAs are a group of RNAs with many different subgroups, but they are not translated into proteins. MicroRNAs (miRNAs) are the most studied type of ncRNAs, and have been found to regulate several pathological processes in MI, including cardiomyocyte inflammation, apoptosis, angiogenesis, and fibrosis. These processes can also be modulated by circular RNAs and long ncRNAs *via* different mechanisms. However, the regulatory role of ncRNAs and their underlying mechanisms in MI are underexplored. Exosomes play a crucial role in communication between cells, and can affect both homeostasis and disease conditions. Exosomal ncRNAs have been shown to affect many biological functions. Tissue-specific changes in exosomal ncRNAs contribute to aging, tissue dysfunction, and human diseases. Here we provide a comprehensive review of recent findings on epigenetic changes in cardiovascular diseases as well as the role of ncRNAs and exosomal ncRNAs in MI, focusing on their function, diagnostic and prognostic significance.

## Introduction

Cardiovascular disease (CVD) is a leading cause of mortality around the world, accounting for 18.6 million deaths in 2019, with coronary heart disease (CHD) being the most common type of CVDs. About 697,000 persons in the United States died from heart disease in 2020 that is one in every five deaths (1, 2). Myocardial infarction (MI), a common manifestation of CHD, is caused by acute or chronic deprivation of nutrients, oxygen, and other important elements necessary for the myocardium survival. MI triggers a chain of severe biochemical and metabolic perturbations in the cardiomyocytes. This imbalance primarily results from acute or chronic ischemia. Cell death in the ischemia area is the initial key event of MI, however a chain of events leads to other heart diseases. Furthermore, MI is associated with several structural and functional consequences leading to permanent heart damage or death (3). This is because cardiomyocytes are terminally differentiated cells and thus have a poor regenerative capacity (4). The infarct size is considered to be the best predictor of future cardiac dysfunction and mortality following acute MI (3).

In biology, epigenetics is a field of study focused on the chemical modification of specific genes or gene-associated proteins that do not involve alterations in the DNA sequence. Growing evidence indicated that epigenetic mechanisms, including DNA methylation, histone modification, and non-coding RNA, are closely related to cardiovascular disease development and regression. Epigenetics mainly regulates cardiovascular disease-related genes function and expression level through DNA methylation, histone modification, and non-coding RNA regulation, thus affecting cardiovascular disease progression. Epigenetic markers are important molecular markers of cardiovascular disease because they occur early in the disease and involve key cardiovascular pathologically related pathways. Most importantly, it can be used as cardiovascular disease biomarkers for cardiovascular disease diagnosis, treatment response prediction and evaluation. As we all know, the pathogenesis of cardiovascular disease remains intricate and complex. Clinically, some cases are still difficult to cure, and the prevalence rate increases with age. Interestingly, because of the reversibility of epigenetic modifications, genes and proteins that control these changes have become new targets for cardiovascular disease treatment. Recently, non-coding RNAs (ncRNAs) and their regulatory functions have aroused great interest (5). There is increasing evidence that ncRNAs, such as microRNAs (miRNAs), circular RNAs (circRNAs), and long non-coding RNAs (lncRNAs) all have regulatory functions and diagnostic value for many types of CVD (6). Regulation of numerous ncRNAs modifies pathophysiological processes in cardiac development, cardiovascular remodeling, and cardiovascular diseases, such as ischemic heart disease, hypertrophy, atherosclerosis, and cardiac fibrosis. MicroRNAs are short-ncRNA molecules ~21–23 nucleotides in length, and are expressed by almost every cell (7). A mature miRNA usually binds to its mRNA 3′ untranslated region by complementary base-pairing, leading to inhibition of translation or mRNA degradation (8–10). From 2008 onwards, scientists around the world reported that circulating miRNAs have good potential as markers for the diagnosis of a variety of diseases (11–13). In this context, emerging evidence has confirmed the diagnostic value of miRNAs in MI (14). Moreover, circRNAs and lncRNAs have also been proposed as biomarkers for MI. Recently, a plethora of papers implicated the key role of exosomal ncRNAs in mediating intercellular and inter-organ communication and CADs development (15, 16).

Herein, we provide a comprehensive review of recent findings on the interplay between ncRNAs and epigenetic machinery in cardiovascular diseases. Furthermore, this paper aims to review and enhance the understanding of the mechanisms and roles of ncRNAs and exosomal ncRNAs in MI, and to provide a basis for clinical diagnosis and new therapeutic strategies for this disease.

## Epigenetic regulation in myocardial infarction

Epigenetic regulatory processes include DNA methylation (DNAm), histone modifications, microRNAs, and lncRNAs (17). The expression of the epigenome differs between various cell types, and modulates single cell gene expression by organizing the nuclear architecture in chromosomes, suppressing or promoting the access of transcription factors to the DNA, and regulating gene expression (18). Epigenetic alterations are involved in the cause of many human diseases, including severe CVD, owing to the role of differential gene regulation in cellular differentiation and biological functions (1, 19, 20). There is much evidence in support of the assertion that epigenetic alterations play a crucial role in a variety of CVDs, such as hypertension, cardiac hypertrophy, heart failure (HF), myocardial I/R damage (21–25). Thus, epigenetics can be considered as a valuable target for the management of myocardial I/R damage.

### Histone modification and histone deacetylases in MI

An important mechanism for epigenetic regulation is histone modification and post-transcriptional modification, and the most frequent histone modifications include acetylation, phosphorylation, ubiquitination, and methylation (26). Researchers reported that histone methylation primarily takes place on arginine and lysine residues, and is mediated by histone demethylase and histone methyltransferase (HMTs) enzymes (27). Histone acetylation by histone acetyltransferases (HATs) is the introduction of acetyl groups into histones, leading to nucleosome relaxation and activation of transcription. The reverse progress is the elimination of acetyl groups from histones by histone deacetylase enzymes (HDACs), which causes the accumulation of nucleosomes and contributes to inhibition of gene transcription. Hence, HATs and HDACs have been likened to writers and erasers (28–30).

The main site of histone methylation in studies focusing on myocardial ischemia/reperfusion (I/R) injury is histone 3 lysine 9 (H3K9). The first confirmed H3K9 methyltransferase was Suv39h1. Since then, several other methyltransferases have been discovered, such as G9a and its related proteins Ctrl4 and Suv39h2. In contrast, PHF8 (PHD Finger Protein 8), JHDM2 (Jmjc domain-containing histone demethylase 2) and JHDM3 enzymes can demethylate H3K9 (27). Yang et al. showed that ischemic or oxidative stress induces Suv39H expression in parallel with the class III protein deacetylase sirtuin 1 (SIRT1) repression, whereas deletion/inhibition of SUV39H ameliorates MI-related damage (31).

According to report by Sung et al., histone methyltransferase G9a plays a significant role in acute myocardial infarction-induced heart failure (32). Flow cytometry analysis clarified the protective impact of G9a inhibitor (i.e., UNC0638) on H9C2 cardiomyocyte cells against H_2_O_2_-indcued apoptosis and oxidative stress. In addition, the combination of G9a inhibitor and erythropoietin (EPO) therapy effectively protected heart against damage from acute myocardial through regulating of several factors. This combined treatment promoted angiogenesis by upregulating the expressions of SDF-1α, VEFG and CXCR4, three angiogenesis indicator. Besides, combination of G9a inhibitor and EPO decreased expressions of apoptotic cleaved caspase 3 and cleaved PARP, fibrotic biomarkers (Samd3, TGF-ß and fibronectin), and DNA-damage marker, and suppressed protein expressions of inflammatory biomarkers [matrix metalloproteinase (MMP)-2 and MMP-9] and anti-oxidative-stress indicators (Sirt1 and Sirt3). The authors also discovered that combining G9a inhibitor and EPO treatment improved autophagy and cell-stress signaling (32).

HDACs are a large family of enzymes consisting of 18 enzymes in humans. They regulate chromatin remodeling and consequent gene transcription mostly by controlling histone acetylation status. Based on sequence homology and cellular localizations, HDACs are divided into four classes: class I (HDAC1, 2, 3, and 8); class II (HDAC4, 5, 6, 7, 9, and 10); class III (SIRT1- 7); and the class IV protein (HDAC11) (33).

Among the HDACs, class IIa (HDAC4, 5, 7 and 9) are thought to be cardiac protective because overexpression of HDAC4, HDAC5, or HDAC9 in cardiac myocytes suppresses expression of a pro-hypertrophy transcription factor, Mef2 (myocyte enhancer factor-2), and reduces stress-induced cardiac hypertrophy (34). Conversely, silencing HDAC5 or HDAC9 leads to an exacerbation of the hypertrophic response to pressure overload (35).

HDAC4 belongs to class II, plays an important role in a number of physiological and pathological processes of the heart. Under normal conditions, HDAC4 activity is very low, while it is activated under pathological conditions, including cardiac injury. In animal models, overexpression of HDAC4 increased myocardial infarct size, on the other hand, inhibition of HDAC4 stimulates regeneration and restoration of cardiac function and reduces myocardial infarction in ischemic HF (36, 37). Zhang et al. created myocyte-specific active HDAC4 transgenic mice to investigate the functional role of activated HDAC4 in regulating myocardial I/R injury. They found that active HDAC4 in the heart is critical to promote myocardial I/R injury. Moreover, delivery of HDAC inhibitor can diminish HDAC4-induced I/R injury (36). Additionally, activated HDAC4-elicited cardiac injury was related the decreased SOD-1 and increased apoptosis and autophagy (36). Recently, Li et al. concluded that HDAC4 silencing can upregulate miR-206 expression, reducing cardiomyocyte apoptosis and inhibiting oxidative stress, and protecting myocardial I/R injury through the mitogen-activated protein kinase kinase kinase 1 (MEKK1)/JNK pathway (38).

In contrast to class IIa HDACs, class I HDACs have shown pro-hypertrophy effects *via* a variety of mechanisms, such as reducing autophagy through activation of mTOR signaling or suppressing the expression of Inpp5f (inositol polyphosphate-5-phosphatase f) and later inhibiting GSK3β (glycogen synthase kinase 3β) signaling, or inhibiting of DUSP5 (dual-specificity phosphatase 5) that negatively regulates ERK1/2-induced cardiac hypertrophy (39).

It was shown that HDAC1 is present in cardiomyocyte mitochondria, and stimulates myocardial damage in the initial phase of reperfusion (40). According to recent researches, overexpression of HDAC2 and HDAC3 induces severe cardiac hypertrophy and hyperplasia, respectively (41, 42). Furthermore, class I HDAC inhibitors diminishes cardiac hypertrophy (43). A number of studies have shown a cardioprotective effect of class III HDACs (e.g., sirtuins) against myocardial I/R damage (44). Overall, it seems that class IIa and class III of HDACs have protective roles not only in heart injury but also in vessel injury, whereas class I HDACs protect against vessel damage but have harmful effects on the myocardium.

Enhancer of zeste homolog 2 (EZH2), an enzymatic subunit of polycomb repressive complex 2 (PRC2), is evolutionarily conserved in many species with comparable structural motifs and domains (45). It is responsible for catalyzing histone H3K27me3 to silence its target gene expression and being involved in various biological functions from cell cycle and cell proliferation to cell differentiation etc. (46, 47). EZH2 represents a major target of ncRNAs inside nucleus (48). Studies showed that in Ezh2-deficient adult hearts, fetal genes are upregulated, causing cardiac hypertrophy (49, 50). In ischemic heart disease (IHD), elevated EZH2 epigenetically represses cardiac sodium channel NaV1.5, which is one of the underlying mechanisms of arrhythmias in IHD patients (51). Recently, Jiao et al. done a study to investigate the therapeutic effects of mesenchymal stem cell-secreted exosomes (MSC-EXO) on myocardial fibrosis after MI (52). They found EZH2 alleviates the cardioprotective effects of MSC-EXO in MI rats through inhibiting HMGA2 (High mobility group AT-Hook 2) expression and disrupting the PI3K/AKT pathway (52).

### DNA methylation and MI

There are several ways to control gene expression, and one example is DNA methylation (31). DNA methylation is the well-studied epigenetic modification of the genome (53). It is essential for cellular reprogramming, normal development, tissue differentiation, and is contribute significantly in many biological processes as well as to the molecular pathology of different disease states (54). DNA methylation occurs when the methyl groups are covalently attached to the 5′ of cytosine in cytosine-guanine (CpG) dinucleotides, found mainly in CpG-rich regions known as CpG islands (CGIs) present in more than half of the promoters of mammalian genes (55). DNA methylation itself modifies transcription binding sites, preventing transcriptional activation and binding of transcription factors (56). Researchers are discovering the mammalian proteins and mediators involved in DNA methylation and demethylation, including DNA methyltransferases (DNMTs), ten-eleven translocation cytosine dioxygenases (TETs), methyl-CpG binding proteins (MeCPs), non-coding RNAs, and transcription factors (57). DNMT1 is an enzyme that tends to maintain nascent DNA methylation marks during mitosis at cytosines that are methylated on the parental strand, termed maintenance methylation. This phenomenon ensures the persistence of programmed DNA methylation patterns across cell generations. *De novo* DNA methyltransferase enzymes DNMT3A and DNMT3B are involved in generating new DNA methylation signatures and are essential for regulating DNA methylation patterns for embryonic or neonatal development (58, 59). DNMT3L as a cofactor is require for *de novo* methyltransferase activity in embryonic stem cells and a positive regulator of DNA methylation at gene bodies of housekeeping genes (60). Proteins, including TET methylcytosine deoxygenase, AID (activation-induced cytidine deaminase), and TDG (thymine DNA glycosylase), are involved in active and passive demethylation, gene activation, embryonic development, and maintenance of adult tissue homeostasis (61–63). TET enzymes catalyze the sequential oxidation of 5-methylcytosine (5 mC) to generate intermediates involved in the conversion of 5 mC to unmodified cytosines and removal of the epigenetic mark, potentially providing the first steps in a pathway for active DNA demethylation (64).

Abnormalities in DNA methylation and hypomethylation across the genome, as well as CpG island hypermethylation have been observed in CVDs (32, 33). Recent advances in epigenetic sequencing have allowed examination of associations between genomic coding, exposure to carcinogens, and disease phenotype. The DNAm epigenetic modification could provide a possible explanation for these relationships (34). It is possible to influence transcription by adding methyl groups to certain DNA nucleotide bases *via* the pre-transcriptional modification known as DNA methylation (35). DNA genetic sequences are essential to the natural evolution and survival of mammalian species governed by specific patterns of gene expression. Epigenetic modifications are dynamic and reversible at the same time, which is important for the regulation of genetic pathways (36). DNA methylation is a dynamic process that reflects the balance between the activity of DNMTs and TETs. The association between mutated genes DNMT3A and TET2 and development of inflammation, atherosclerosis, and heart failure has been reported (65–67). Numerous studies have focused on DNAm pattern alterations and measuring differentially methylated regions in both normal and abnormal development (37).

A number of studies have investigated DNA methylation profiles in CHD and MI, suggesting their significance as a diagnostic marker (68, 69). One study showed notable enhanced DNA methylation levels in CHD patients (70). Additionally, in a pilot study by Ma et al. for providing useful DNA methylation profiles to serve as biomarkers for detecting early-phase atherosclerosis (AS), a total of 300 persons were recruited (150 AS patients and 150 healthy subjects) for peripheral blood DNA methylation analyses at 12 gene promoter loci using nested methylation-specific polymerase chain reaction (71). Based on the test set, promoter methylation of acetyl Acetyl-CoA acetyltransferase 1 (ACAT1) was decreased whereas ATP binding cassette subfamily A member 1 (ABCA1) and tissue inhibitor of metalloproteinase-1 (TIMP1) were significantly increased in AS compared the matched controls. Thus, they suggested that methylation of the three-gene panel (TIMP1, ABCA1, and ACAT1) could serve as a valuable, high sensitivity, and reliable biomarker for the early detection of AS (71). Perinatal nicotine exposure in mice was shown to increase myocardial I/R damage, which was found to be mediated by DNA hypermethylation and increased DNMT3 expression. Conversely, the administration of the DNA methylation inhibitor 5-aza-2′-deoxycytidine (5-Aza) was able to ameliorate myocardial injury. In conclusion, the authors found out that nicotine-induced myocardial I/R damage was mediated by DNA methylation in perinatal mice (72).

## Non-coding RNAs and myocardial infraction

More than 90% of the human genome is composed of genes that do not encode protein. Nevertheless, these genes show considerable transcriptional activity, and code for a wide variety of ncRNAs with biological regulatory functions (73).

**Figure 1:**
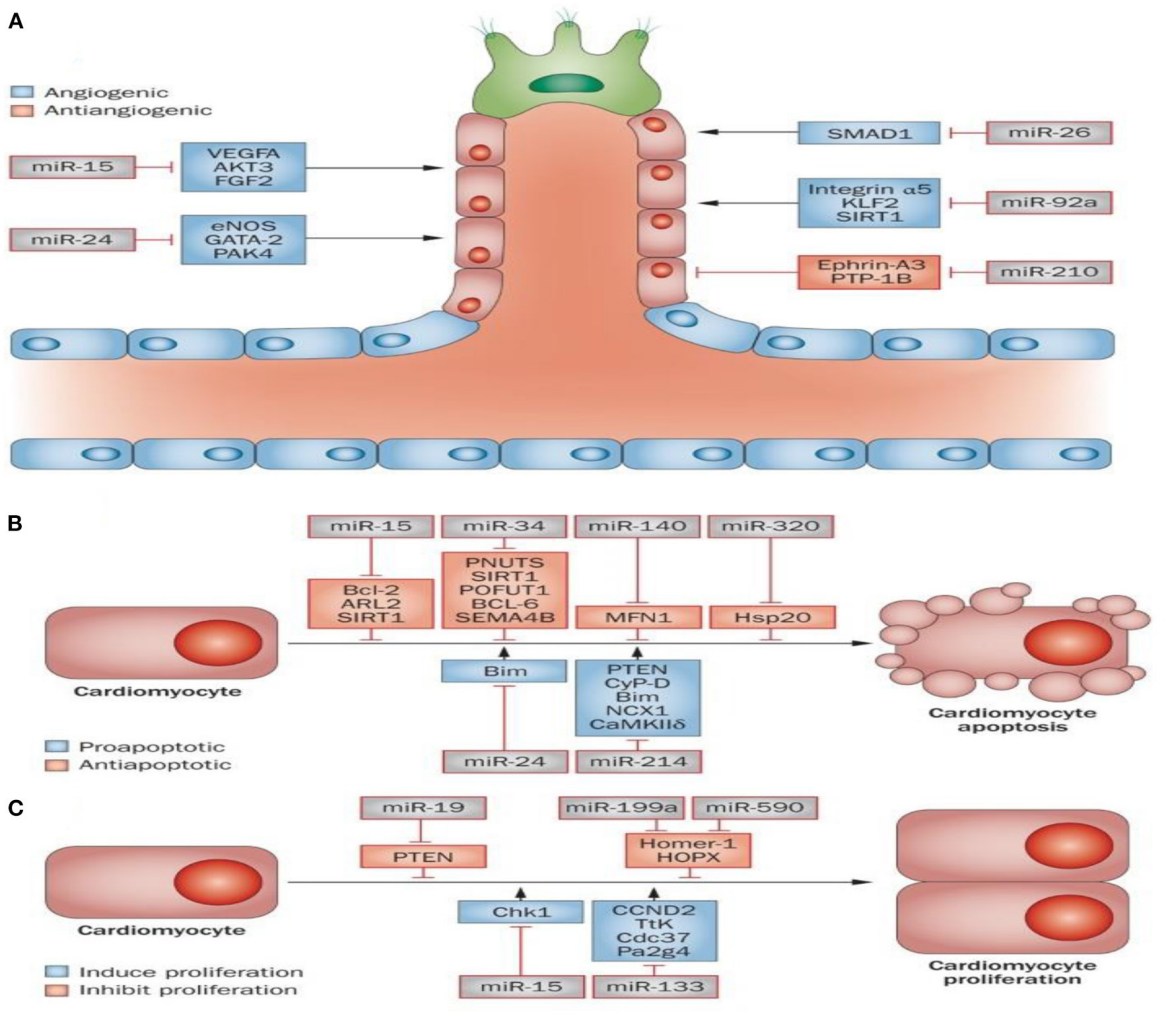
Role of microRNAs in MI. Some miRNAs are known to be implicated in cardiac muscle angiogenesis **(A)**, cardiac cell survival, and proliferation **(B,C)**. Angiogenesis is pivotal for the delivery of oxygen and essential nutrients to heart muscle. Angiogenesis can be activated or repressed by a number of microRNAs. For instance, miR-15, miR-24, miR-26, and miR-92a all repress angiogenesis through inhibition of endothelial-cell functions. In contrast, miR-210 prompts angiogenesis *via* inhibiting several antiangiogenic factors **(A)**. A number of miRNAs have been identified to be involved in the apoptosis and survival of cardiac cells. Apoptosis inhibition or an increase in survival signals promotes cardiac regeneration. The miR-15 family (miR-320, miR-34, and miR-140) can serve as pro-apoptotic factors, while miR-24 and miR-214 can act as anti-apoptotic factors (B). The proliferation of cardiac cells is limited; however, cardiac regeneration can increase following stimulation of proliferation. It has been shown that miR-19, miR-199a and miR-590, can promote proliferation of cardiac cells, while both miR-15 and miR-133 inhibit cardiac cell proliferation **(C)**. This figure adapted from Boon and Dimmeler (76).

As discussed above, ncRNAs include three main classes: miRNAs, lncRNAs, and circRNAs. lncRNAs contain more nucleotides compared to miRNAs (>200 nucleotides vs. 21–23 nucleotides), which is the primary difference between them. MiRNAs were shown to modulate gene expression by inhibiting messenger RNA (mRNA) translation and causing mRNA degradation (6, 74, 75). It has been reported that miRNAs can affect myocardial angiogenesis and cardiomyocyte survival and proliferation by regulation of target gene expression (Figures 1A–C).

CircRNAs are mostly found in eukaryotic cells. Unlike normal linear RNA molecules, the 3-cap and 5-tail of circRNAs are covalently joined to form a closed loop. CircRNAs are characterized by plentiful amounts, tissue specificity, and evolutionary conservation. CircRNAs can act as a competitive endogenous RNA (ceRNA) and a sponge that binds to complementary miRNA sequences to moderate gene expression (77, 78).

## MicroRNAs and myocardial infarction

MiR-431 has been shown to stimulate the regeneration and differentiation of axons and skeletal muscles by targeting Smad4 (79). Emerging evidence has shown that miR-431 plays an important role in several diseases, including but not limited to hepatocellular carcinoma (80) and colorectal cancer (81). The possible underlying mechanism was suggested to be hypoxia-induced apoptosis (82). Nevertheless, the involvement of miR-431 in cardiac hypoxia/reoxygenation (H/R) is still unclear. It is known that autophagy and cell apoptosis are two important processes involved in H/R and MI (83). Autophagy-related 3 (ATG3) is an E2 enzyme essential for the lipidation of LC3 during autophagy (84). There is evidence that ATG3 is associated with autophagy in several diseases, including breast cancer (85), hepatocellular carcinoma (86), and chondrocyte disturbance (87), however, the role of miR-431 in myocardial damage is not yet understood. Zhou et al. tried to clarify the mechanism of how miR-431 affected apoptosis and autophagy of cardiomyocytes in H/R. They found that H/R was associated with decreased cell viability, increased apoptosis, and lower miR-431 expression in human cardiomyocytes. Inhibition of miR-431 decreased cell viability and promoted cell apoptosis. Additionally, it was shown that miR-431 inhibited ATG3 expression by targeting the 3'-untranslated region of ATG3 mRNA. Exogenous ATG3 overexpression was able to reverse the effects of miR-431 up-regulation on cell survival and apoptosis in H/R-treated human cardiomyocytes. H/R treatment stimulated autophagy in human cardiomyocytes, which was significantly decreased after transfection with a miR-431-mimic. Their findings indicated that miR-431up-regulation could regulate ATG3 expression, and ameliorate H/R-stimulated myocardial injury (88).

Treatment with imatinib mesylate and doxorubicin increased miR-205 expression in the plasma and heart of mice (89). Moreover, miR-205 downregulation significantly decreased myocardial apoptosis in rats with chronic heart failure (90). Cheng et al. sought to understand how Remifentanil (a short-acting synthetic opioid analgesic drug) exerted its protective effects on myocardial I/R damage. The authors found that remifentanil was able to enhance cell survival and reduce apoptosis of H/R-treated cardiomyocytes. Increased miR-205 expression and reduced PINK1 (PTEN induced putative kinase 1) expression was observed following H/R treatment. Preconditioning with remifentanil decreased miR-205 and increased PINK1 expression. Additionally, miR-205 up-regulation decreased the expression of PINK1 and reversed the remifentanil-stimulated increase in cell viability and reduction of apoptosis in H/R-treated cardiomyocytes. The investigators observed that the use of a miR-205 antagomir was associated with a better remifentanil-induced reduction of lactate dehydrogenase (LDH) activity and lower infarct size in rats with I/R injury. Ultimately, miR-205 may be the reason for the protective effect of remifentanil on myocardial I/R damage, making it a possible target for the management of MI (91).

It was reported that miR-147 could inhibit apoptosis in L6 myoblasts subjected to cyclic mechanical stretching (92). MiR-147 downregulation was associated with inhibition of proliferation in gastric cancer cells (93). Nonetheless, the role of miR-147 in MI remains largely unknown. Wu et al. designed an *in vivo* study to evaluate the function of miR-147 in MI, and the mechanism of action (94). Quantitative reverse transcription-polymerase chain reaction (qRT-PCR) was used to measure the expression of Bax mRNA, miR-147, and Bcl-2 mRNA. Inflammatory cytokines including interleukin (IL)-6, IL-β, TNF-α, as well as lactate dehydrogenase (LDH) were measured by enzyme-linked immunosorbent assay (ELISA). Additionally, the MTT [3-(4, 5-dimethylthiazol-2-yl)-2, 5-diphenyl tetrazolium bromide] assay was employed to measure cell viability. Cell apoptosis was measured by terminal dexynucleotidyl transferase (TdT)-mediated dUTP nick end labeling (TUNEL). Additionally, *in vivo* cardiac function was evaluated by echocardiography. Western blotting was employed to measure the expression of homeodomain interacting protein kinase 2 (HIPK2). Results indicated the lower levels of miR-147 in the rat MI model and also in H9c2 cells treated with H_2_O_2_. Moreover, H_2_O_2_ treatment was associated with higher levels of inflammatory cytokines and caused apoptosis in H9c2 cells. These effects were abrogated by miR-147 up-regulation. MiR-147 up-regulation reversed the increased Bax expression and reduced the Bcl-2 expression in H9c2 cells induced by H_2_O_2_. Furthermore, miR-147 up-regulation was associated with better cardiac function and lower serum LDH values in MI rats. TargetScan analysis demonstrated that HIPK2 could bind to miR-147. Additionally, miR-147 up-regulation was able to inhibit HIPK2 expression (94). The findings of this study provide the postulate that lower levels of miR-147 can be expected in patients with MI, while up-regulation could inhibit myocardial apoptosis and inflammation, and improve cardiac function through regulating HIPK2 (94).

MiR-130 can worsen myocardial injury following MI by targeting peroxisome proliferator-activated receptor gamma (PPAR-γ). In H9c2 cells, miR-130 by targeting PPAR-γ aggravates acute MI-induced injury. *In vivo* experiments confirmed that miR-130 downregulation promotes PPAR-γ-mediated cardioprotective effects by suppressing NFκB-mediated inflammation and TGF-β1-mediated myocardial fibrosis (85). Pan et al. designed a study to evaluate the diagnostic value of miR-130 in patients with MI. The plasma expression of miR-130 in patients suffering from MI was measured by qRT-PCR. The plasma value of cardiac troponin I (cTnI) and creatine kinase-MB (CK-MB) was examined by ELISA. The authors evaluated the diagnostic value of miR-130 using a receiver operating characteristic (ROC) curve. The plasma values of miR-130, CK-MB, and cTnI in the MI group were significantly higher relative to the control group. The peak of miR-130 expression occurred 6 h after MI onset and the peak was earlier compared to CK-MB and cTnI. A positive correlation was found between miR-130 (6 h after MI onset) and the values of CK-MB and cTnI (12 h after MI onset). The optimal cut-off point was estimated as 1.58 ng/mL with a sensitivity of 82.5% and specificity of 77.5%. The area under the curve (AUC) was measured to be 0.922. These findings suggest that miR-130 could be utilized as a biomarker for MI diagnosis (95).

According to evidence, in patients with HF, the cardiac microRNA-132-3p level is increased in cardiac tissue in response to cardiomyocyte stress (96). It mechanistically drives cardiac remodeling processes leading to heart failure through downregulating the expression of the anti-hypertrophic, pro-autophagic transcription factor Forkhead box O3 (FOXO3) and also inhibiting the expression of genes involved in intracellular calcium handling and contractility (97). As a result, miR-132 appears to be a potentially promising molecular pathophysiological target in the treatment of HF. In this regard, a randomized, placebo-controlled, double-blind, phase 1b dose-escalation study (NCT04045405) was conducted to evaluate the safety, pharmacokinetics, and exploratory pharmacodynamic effects of CDR132L; a synthetic antisense oligonucleotide (ASO) inhibitor, in heart failure patients (98). Based on the results, CDR132L is safe and tolerable, has confirmed linear plasma pharmacokinetics and shows no signs of accumulation, and improves cardiac function (98). Currently, in a randomized, parallel, 3-arm, placebo-controlled phase II clinical trial (NCT05350969) the safety and efficacy of CDR132L in patients with reduced left ventricular ejection fraction (≤45%) after MI is also under investigation (33).

It has been found that miR-204 could affect several physiological and pathological processes, including apoptosis, cell proliferation, and inflammation. Additionally, its capability to affect signaling pathways and their downstream effectors has been demonstrated (99, 100). Therefore, there is a possibility that miR-204 may participate in the process of MI by modulating different pathological responses.

Wang et al. evaluated the effect of miR-204 in rats with MI by targeting the silent information regulator 1 (Sirt1)/p53 signaling pathway. They divided 36 rats into three equal groups, including the sham-operated group, the MI model group, and the miR-204 mimic MI group. The rats in the sham-operation group merely underwent thoracotomy, without any MI injury. MI injuries were created in the MI model group and the miR-204 mimic group and treated with normal saline or miR-204 mimic, respectively. Histological staining revealed a normal morphology in the sham-operated group and severe myocardial tissue damage in the model group. The damage was less in the miR-204 mimic group relative to the MI model group. The authors found a higher Caspase-3 expression in cardiac tissue of the model group and miR-204 mimic group relative to the sham-operated group. The miR-204 level was significantly higher in the model group compared to the miR-204 mimic group. It was shown that Sirt1 (Sirtuin 1) could act as the target gene of miR-204. Higher expression of Sirt1 and p53 was detected in the model group and the miR-204 mimic group compared to the sham-operation group. However, their expression level was notably lower in the miR-204 mimic group compared to the model group. Regarding miR-204 expression, both the miR-204 mimic group and the model group had lower expression; nonetheless, its expression was considerably higher in miR-204 mimic group relative to the model group. A higher apoptosis rate was detected in both the model group and the miR-204 mimic group compared to the sham-operation group. But apoptosis was lower in the miR-204 mimic group compared to the model group. They concluded that miR-204 was able to decrease the apoptosis rate in MI *via* targeting the Sirt1/p53 signaling pathway (101) (Figure 1, Table 1).

**Table 1:** Reports of changes in miRNA expression in laboratory models and patients with MI.

**MicroRNAs**	**Expression**	**Target**	**Mechanism**	**Model (*in vitro, in vivo*, human)**	**Tissue, blood, cell**	**References**
miR-204	Down	LC3-II	Regulated autophagy	*In vivo*	Tissue	(102)
miR-122	Down	–	–	Human*, in vivo*	Tissue, blood	(103)
miR-519e-5p	Down	–	–	Human, *in vitro*	Blood, H9c2, Raw 264.7, and HUVEC cells	(104)
miR-150	Down	Egr2, p2x7r	Repressed apoptosis and inflammation	*In vivo, in vitro*	Tissue, and HL-1, H9c2, MCEC, VSMC, and NRVC lines	(105)
miR-132-5p	Down	–	–	Human	Blood	(106)
miR-1291, miR-217, miR-455-3p, miR-566	Down	–	–	Human	Blood	(107)
miR-147	Down	HIPK2	Inhibited inflammation and apoptosis, improved cardiac function	*In vitro, in vivo*	Tissue, H9c2 cells	(94)
miR-1, miR-92a, miR-99a, miR-223	Down	–	–	*In vitro*, human	Tissue, blood	(108)
miR-873	Down	RIPK1, RIPK3	Regulated programmed necrosis	*In vitro*	Cardiomyocytes	(109)
miR-206	Down	PTP1B	Reduced myocardial infarct size and cardiomyocytes apoptosis	*In vivo, in vitro*	Tissue	(110)
miR-378	Down	caspase-3	Enhanced cell viability, reduced lactate dehydrogenase release, inhibited apoptosis and necrosis	*In vivo, in vitro*	Tissue	(111)
miR-1915	Down	–	–	Human	Blood	(112)
miR-126-3p, miR-26a-5p, miR-191-5p	Down	–	–	Human	Blood	(113)
miR-125b, miR-320b	Down	–	–	Human	Blood	(114)
miR-500, miR-532-3p	Down	–	–	*In vivo*	Tissue	(115)
miR-423-5p, miR-30d	Up	–	–	Human	Blood	(116)
miR-375	Up	PDK-1	Inhibited proliferation and tube formation, and enhanced apoptosis	*In vitro, in vivo*	Tissue, BMPACs	(117)
miR-208a	Up	cAMP-PKA	Influenced the cAMP-PKA signaling pathway	*In vitro, in vivo*	Tissue, blood	(118)
miR-22-5p, miR-150-3p	Up	–	–	Human	Blood	(106)
miR-126-3p	Up	–	–	Human	Blood	(119)
miR-19b-3p, miR-134-5p, miR-186-5p	Up	–	–	Human	Blood	(120)
miR-17-5p, miR-126-5p, miR-145-3p	Up	–	–	Human	Blood	(121)
miR-122-5p	Up	–	–	Human	Blood	(122)
miR-130	Up	–	–	Human	Blood	(95)
miR-21	Up	Smad7	Promoted cardiac fibrosis	*In vitro, in vivo*	Tissue, Primary cardiac fibroblasts	(123)
miR-16	Up	β2-AR	Reduced cell viability, increased apoptosis	*In vitro, in vivo*	Tissue, NRVCs	(124)
miR-125b-5p, miR-30d-5p	Up	-	-	Human	Blood	(125)
miR-124	Up	-	-	Human	Blood	(126)
miR-144-3p	Up	PTEN	Promoted cell proliferation, migration, and collagen production	*In vitro, in vivo*	HCFs	(127)
miR-337, miR-34b	Up	–	–	*In vivo, in vitro*	Tissue	(128)
miR-424-5p	Up	–	–	Human	Blood	(129)
miR-205	Up	PINK1	–	*In vivo, in vitro*	Tissue, primary cardiomyocytes	(91)
miR-486, miR-150	Up	–	–	Human	Blood	(130)
miR-320a, miR-660-5p	Up	–	–	Human	Blood	(131)
miR-197, miRNA-223	Up	–	–	Human	Blood	(132)
miR-140	Up	–	–	Human	Blood	(133)
miR-181a	Up	–	–	Human	Blood	(134)
miR-181c	Up	–	–	Human	Blood	(112)
miR-25-3p, miR-221-3p	Up	–	–	Human	Blood	(135)
miR-210	Up	HIF1α	Decreased cardiac function	*In vivo*, human	Tissue	(136)
miR-486-3p	Up	–	–	Human	Blood	(137)
miR-3559-5p, miR-499, miR-21	Up	–	–	*In vivo*	Tissue	(115)

## Long non-coding RNAs and myocardial infarction

LncRNAs have important regulatory roles in cardiac cell growth, cell survival, and fibrosis in MI. They exert their effects on heart disease through affecting epigenetic gene modifications, transcriptional, and post-translational regulation *via* direct binding to proteins, miRNAs, and other lncRNAs (138). Although the number of discovered lncRNAs has increased over the years, there is still not much information about the mechanisms of action and the functions performed by these biomolecules. One of the reasons for this delay is that many lncRNAs show unique temporal and spatial expression patterns (139). Furthermore, unlike microRNAs and mRNAs, most lncRNAs have poor sequence conservation among different species, which makes it difficult to obtain functional information and mechanisms of action of the vast majority of lncRNAs and, consequently, translate findings from animal models to humans (139, 140). Besides, the link between basic research and clinical trials in animal models. Therefore, the non-conservation of the nucleotide sequence of lncRNAs among different species is a big and influential challenge, because it makes it difficult to transfer the findings obtained in preclinical studies to humans (141). Hence, clinical trials are limited to working with only those lncRNAs that have human counterparts.

In addition to the issues listed above, the secondary and tertiary structures of lncRNAs must be explored, as they may have structural homologs in other species (142, 143). Finally, drug delivery to the target lncRNA of interest remains a difficulty (144). Even though there are still many challenges to utilizing lncRNAs as a therapeutic approach in CVDs, lncRNAs are promising candidates for the clinic, and due to their specific expression pattern associated with various pathologies, they are recognized as a tool with great application power in personalized medicine (140). In the following, we will discuss some studies about the role of lncRNAs in heart disease and their mechanisms of action.

In an *in vitro*/*in vivo* study, Su et al. reported that hypoxia increased the expression of the lncRNA taurine upregulated gene 1 (TUG1), then TUG1 sponged miRNA-132-3p and activated HDAC3, which in turn induced several targets protective gene, stimulated intracellular ROS accumulation, and exacerbated acute MI injury (145).

It was discovered that the lncRNA X inactive specific transcript (XIST) was involved in the inactivation of chromosome X in female cells during embryonic development (146), and it was also overexpressed in several malignancies, such as lung and gastric cancer (147, 148). High levels of XIST were observed in a rat model of acute MI, and its knockdown was shown to suppress cardiomyocyte apoptosis *via* downregulating miR-449 (149).

Zhou et al. examined the function and underlying mechanism of lncRNA XIST in hypoxia-stimulated injury in H9c2 cells. Cell viability, invasion, migration, and apoptosis were assessed utilizing MTT, transwell, and flow cytometry assays, respectively. Gene expression was measured using Western blotting or qRT-PCR. XIST up-regulation was found in H9c2 cells after hypoxia-stimulated injury, and its knockdown ameliorated the cell damage. XIST overexpression enhanced the expression of B-cell lymphoma 2-associated X (Bax) by binding to miR-150-5p. Thus, it was concluded that XIST was able to protect cardiomyocytes from hypoxia-stimulated damage by modulating the miR-150-5p/Bax axis, suggesting that XIST could a target for MI treatment (150).

The lncRNA called “smooth muscle and endothelial cell-enriched migration/differentiation-associated lncRNA” (SENCR) is located on chromosome 11, and is expressed in human vascular cells (151). There is much evidence in support of the role of SENCR in inhibiting the proliferation and migration of human aortic-vascular smooth muscle cells. SENCR could reduce the formation and progression of atherosclerotic lesions by modulating the miR-4731-5p/FOXO3a signaling pathway (152).

Chen et al. designed a study to evaluate the role and underlying mechanism of SENCR on H/R-stimulated apoptosis in cardiomyocytes. qRT-PCR was employed to quantify the expression of SENCR in the serum of the case group (MI patients) and the control group (non-MI patients with chest pain). Cell viability, cell apoptosis, and inflammatory responses were assessed by MTT, flow cytometry, and ELISA, assays. The luciferase reporter assay was employed to identify the mechanism of SENCR. Downregulation of SENCR was detected in MI patients compared to the control group, and its expression was negatively correlated with cTnI and CK-MB levels in MI patients. H/R-stimulated apoptosis and inflammation were attenuated by SENCR up-regulation. SENCR up-regulation was associated with the downregulation of miR-1 expression. SENCR up-regulation also decreased apoptosis, mitigated inflammatory responses, and increased cell survival in cardiomyocytes. SENCR was shown to be able to reverse H/R myocardial damage *via* downregulating miR-1. A significant correlation between SENCR expression and the clinicopathological characteristics of MI patients was found (153).

The lncRNA plasmacytoma variant translocation 1 (PVT1), which is located on chromosome 8q24, is a potential oncogene in several malignancies (154). Furthermore, an *in vivo* study showed that PVT1 was remarkably overexpressed in the hypertrophic mouse heart, and its inhibition was associated with a smaller cardiomyocyte size in Angiotensin II-treated cardiomyocytes, suggesting a crucial role of PVT1 in cardiomyocyte hypertrophy (155). However, its role in other cardiac diseases is not yet clear.

Ouyang et al. established a study to assess the involvement of lncRNA PVT1 in H/R-treated AC16 cardiomyocytes (156). They found out that treatment decreased cell survival and enhanced apoptosis, and its suppression ameliorated the H/R damage and besides, decreased excessive autophagy. Additionally, the authors demonstrated that it may act as a ceRNA to sponge miR-186 in AC16 cells, and rescue research revealed that prohibition of miR-186 was able to suppress the effects of PVT1 suppression in H/R-treated AC16 cells. Thus, PVT1 can be suggested as a valuable target for myocardial I/R injury treatment (156).

Testis-specific transcript Y-linked 15 (TTTY15) is a transcription-mediated chimeric lncRNA with key regulatory functions in some CVDs (157, 158). Bioinformatics and experimental analysis revealed that TTTY15 could promote myocardial cell injury by regulating the miR-98-5p/C-reactive protein (159).

Chen et al. aimed at investigating the role of lncRNA TTTY15 in myocardial I/R damage and its putative interaction mechanisms with miR-374a-5p. *In vivo* and *in vitro* experiments revealed enhanced TTTY15 expression in myocardial I/R damage. Besides, TTTY15 inhibited autophagy as well as myocardial I/R damage *via* modulating the expression of miR-374a-5p. TTTY15 modulated miR-374a-5p expression, thereby regulating FOXO1 expression and autophagy in myocardial I/R damage. Furthermore, TTTY15 inhibition ameliorated *in vivo* myocardial I/R injury. According to their findings, they concluded that TTTY15 can be taken into account as a precious target for myocardial I/R injury treatment (160).

RNA Component of Mitochondrial RNA Processing Endoribonuclease (RMRP), a recently explored lncRNA consisting 267 nucleotides, has been demonstrated to exhibit carcinogenic effects in a variety of malignancies, including gastric cancer (161), glioma (162), and lung cancer (163). Steinbusch et al. pointed out that RMRP has a pivotal role in chondrocyte hypertrophy and it can contribute to cartilage-hair hypoplasia (164), suggesting the mandatory function of this lncRNA in several diseases. Of note, dysregulation of RMRP has been observed in patients suffering from ischemic heart failure (165). However, there is a scarcity of data regarding whether RMRP is dysregulated in MI and myocardial I/R damage.

Kong et al. sought to clarify the roles of RMRP and its mechanism in myocardial I/R damage (166). To induce myocardial damage, the H9c2 cardiomyocytes were cultured under hypoxia. Moreover, a rat myocardial I/R damage model was established by subjection to 60 min ischemia and 24 h reperfusion. The authors declared that RMRP up-regulation was associated with worsen hypoxia-induced damage in cardiomyocytes. A negative correlation between RMRP and miR-206 was also found and RMRP up-regulation aggravated hypoxia damage *via* downregulation of miR-206. Additionally, it was shown that miR-206 exerted its effects on hypoxia damage by targeting autophagy related 3 (ATG3). Also, RMRP up-regulation was associated with activation of PI3K/AKT/mTOR signaling pathway in hypoxia-treated H9c2 cells *via* regulation of miR-206/ATG3. In summary, it can be concluded that RMRP overexpression has the potential to aggravate myocardial injury through the downregulation of miR-206 and consequently overexpression of ATG3. In the setting of myocardial I/R damage, PI3K/Akt/mTOR pathway activation serves as a central downstream mediator of the RMPR/miR-206/ATG3 axis (166).

Clarifying the mechanisms and pathways behind exercise-stimulated adaption will offer new therapeutic targets that can be utilized as treatments for CVDs (140). The preventive and therapeutic effects of exercise on CVDs have been well-established, hence, it can be considered another strategy aimed at regulating the expression of lncRNAs (140). Evidence demonstrated that aerobic exercise can decrease cardiomyocytes' apoptosis and heart fibrosis areas following MI *via* modulating the expressions of myocardial infarction associated transcript (MIAT), H19, and lncRNA GAS5. In addition, exercise can prohibit the expression of MIAT, known pro-fibrotic lncRNA in the MI settings, which exerts its impacts by sequestering miRNA-24 and provoking the TGF-beta signaling pathway (140).

In neonatal and adult mice, suppression of CAREL (cardiac regeneration-related long non-coding ribonucleic acid) enhanced heart function and was associated with higher cell proliferation and regeneration (167). CRRL (cardiomyocyte regeneration-related lncRNA), like CAREL, is a negative regulator of cardiomyocyte proliferation and cardiac repair. CRRL achieves these effects by binding to miR-199a-3p, inhibiting its activity and boosting levels of its target Hopx (168).

lncRNA ZNFX1 antisense 1 (ZFAS1) is made from a snoRNA host gene. It is one of the lncRNAs involved in cardiac hypertrophy, as its knockdown rescued contractile dysfunction (169, 170). Sarcoplasmic/endoplasmic reticulum Ca2+ ATPase 2a (SERCA2a) is a key protein in the Ca2+ cycle of HF so its dysregulation is a hallmark of HF (171). LncRNA ZFAS1 as a SERCA2a inhibitor binds to SERCA2A protein and disrupts its activity, leading to changed Ca2+ transient and intracellular Ca2+ load in the heart and contractile dysfunction in an animal model of MI (170) (Table 2).

**Table 2:** Reports of changes in lncRNA expression in laboratory models and patients with MI.

**LncRNA**	**Expression**	**Target**	**Mechanism**	**Model (*In vitro*, *in vivo*, human)**	**Tissue, blood, cell**	**References**
SARRAH	Down	NRF2, GPC6, PDE3A, ITPR2, PARP8, SSBP2	Decrease apoptosis	*In vitro, in vivo*	Tissue	(172)
SENCR	Down	miR-1	Alleviated apoptosis and inflammatory response	Human, *in vitro*	Blood	(153)
CARL	Down	miR-539	Suppressed mitochondrial fission and apoptosis	*In vivo, in vitro*	Tissue	(173)
NONMMUT036355, NONMMUT003691, NONMMUT034297, NONMMUT073076, KnowTID_00004703	Down	–	–	*In vivo*	Tissue	(174)
CAIF	Down	p53	Suppressed autophagy	*In vivo, in vitro*	Tissue	(175)
Dancr	Down	miR-6324	Inhibited apoptosis, enhanced autophagy	*In vitro*	H9c2 cell line	(176)
UCA1	Down	miR-128	Alleviated autophagy	*In vivo, in vitro*	Tissue	(177)
UCA1	Down	miR-128	Alleviated autophagy	*In vivo, in vitro*	Tissue	(177)
UCA1	Down	miR-128	Alleviated autophagy	*In vivo, in vitro*	Tissue	(177)
FAF	Down	FGF9	Inhibited apoptosis	*In vivo, in vitro*	Tissue, cardiomyocytes	(178)
N1LR	Down	TGF-β1, Col1a1, Col3a1, α-SMA	Alleviated apoptosis, inflammation reaction and fibrosis	*In vitro, in vivo*	Tissue, H9c2 cell line	(179)
NR_047662.2, uc002ddj.1	Down	–	–	Human	Blood	(180)
MIRT1, MIRT2	Up	–	–	*In vivo*	Tissue	(181)
NONMMUT013316, NONMMUT030245, NONMMUT065582, KnowTID_00006395	Up	–	–	*In vivo*	Tissue	(174)
NONMMUT022554	Up	PI3K-Akt	–	*In vivo*	Tissue	(174)
NONMMUT023529, NONMMUT022555, NONMMUT72211, KnowTID_00006493	Up	–	–	*In vivo*	Tissue	(174)
TUG1	Up	miR-142-3p	Increased apoptosis and autophagy	*In vivo, in vitro*	Tissue	(182)
PVT1	Up	miR-186	Increased apoptosis and autophagy	*In vitro*	AC16 cells	(156)
NEAT1	Up	miR-378a-3p	Promoted cell proliferation and migration, regulated expression of autophagic factors	*In vivo, in vitro*, human	Tissue, blood	(183)
TTTY15	Up	miR-374a-5p	Suppressed autophagy	*In vivo, in vitro*	Tissue, H9c2 and HL-1 cells	(160)
HRIM	Up	–	Suppressed autophagy	*In vivo, in vitro*	Tissue, H9c2 cell line	(184)
AK088388	Up	miR-30a	Promoted autophagy	*In vitro*	HL-1 cell line	(185)
FOXD3-AS1	Up	NF-κB	Enhanced apoptosis and autophagy	*In vitro*	H9c2 cell line	(186)
AK139328	Up	miR-204-3p	Promoted autophagy	*In vivo, in vitro*	Tissue, cardiomyocyte	(187)
MHRT	Up	–	Decreased apoptosis	*In vitro*, human	Blood, cardiomyocytes	(188)
NRF	Up	miR-873	Increased necrosis	*In vitro, in vivo*	Tissue, cardiomyocytes	(109)
THRIL	Up	miR-99a	Decreased cell viability, migration and invasion, increased apoptosis	*In vitro*	H9c2 cell line	(189)
XIST	Up	miR-150-5p	Inhibited apoptosis	*In vitro*	H9c2 cell line	(150)
TTTY15	Up	miR-455-5p	Decreased cell migration and invasion, increased apoptosis	*In vitro*	HCMs	(158)
NONMMUT032513, NONMMUT074571	Up	ZEB1	–	*In vivo*	Tissue	(190)
ENST00000581794.1, ENST00000509938.1	Up	–	–	Human	Blood	(180)

## Circular RNAs and myocardial infarction

Luo et al. examined circRNA expression throughout the stages of MI progression in an animal model of MI, and in cardiomyocytes with H/R injury *in vitro* (191). They found high expression of circRNA PVT1 (circPVT1) in both MI tissues and H/R-treated cardiomyocytes. The effect of circPVT1 on cardiac function and cardiomyocyte viability was investigated using loss-of-function assays. Echocardiography was employed to assess cardiac function seven days after MI. Lower circPVT1 expression substantially reduced the infarct size by 60% and avoided the MI-induced decrease in fractional shortening (FS) and ejection fraction (EF). Laboratory findings demonstrated that silencing of circPVT1 was associated with higher cell survival and proliferation, and decreased apoptosis. A significant association between circPVT1 expression and both miR-200a and miR-125b was found. The authors suggested that circPVT1 might sponge miR-200a and miR-125b by acting as a ceRNA (191). Overexpression of miR-125b and miR-200a partly abolished the effects of circPVT1 on cardiomyocyte function. Moreover, the authors reported that the circPVT1/miR-125b/miR-200a axis was able to regulate the SIRT7, p53/TRAF6, PDCD4, and Keap1/Nrf2, signaling pathways. In summary, their findings suggested that circPVT1 exerted a cardioprotective effect against MI and H/R damage by inhibiting apoptosis mediated by miR-200a and miR-125b (191).

CircROBO2 is a recently discovered circRNA, but its role in MI is not fully understood. Chen et al. investigated the pathophysiology of circROBO2 in MI (192). Accordingly, Western blotting and qRT-PCR were employed to assess the expression levels of circROBO2, TRADD, and miR-1184 in MI and sham-operated mouse models at protein and mRNA levels, respectively. Luciferase reporter gene analysis and RNA immunoprecipitation (RIP) were used to investigate the association between miR-1184, circROBO2, and TRADD. Flow cytometry was used to verify the involvement of circROBO2, miR-1184, and TRADD in myocardial cell apoptosis. To investigate the effects of circROBO2 on myocardial damage, researchers used ultrasound echocardiography, serum LDH, serum CK-MB, MI area and measured myocardiocyte apoptosis (192). Compared to the control group, miR-1184 expression levels were significantly lower in the MI group; however, circROBO2 and TRADD expression levels were substantially higher. After overexpression of TRADD, circROBO2 behaved like a sponge for miR-1184. Furthermore, miR-1184 up-regulation increased the protective effect of circROBO2 knockdown by inhibiting TRADD expression. The authors concluded that circROBO2 knockdown lowered cardiomyocyte apoptosis by boosting miR-1184 expression, and reducing TRADD expression in the myocardium after MI (192).

Dysregulation of CircHIPK3 has been detected in several diseases including but not limited to diabetes, malignancies, preeclampsia, and retinal vascular dysfunction (193). It was shown that circHIPK3 suppressed proliferation and induced apoptosis of cardiomyocytes with ischemic-reperfusion injury via binding to miR-124-3p (194). Upon hypoxic injury, cardiomyocyte-derived exosomal circHIPK3 plays a crucial role in maintaining cardiac microvascular endothelial cell function by targeting the MIR29A/IGF-1 pathway and *via* modulating the miR-29a/IGF-1 axis (16).

Wu et al. examined the underlying ceRNA network involving circHIPK3 in MI (195). A hypoxic model was used to establish MI *in vivo*, then, the expression levels and association between miR-93-5p, circHIPK3, and Rac1 were evaluated. Gain and loss-of-function experiments were used to assess the ceRNA mechanism (195). CircHIPK3 suppression was associated with decreased myocardial apoptosis, a lower infarct size, myocardial collagen deposition, and improved cardiac function. CircHIPK3 sponged miR-93-5p, while miR-93-5p targeted Rac1. Up-regulation of MiR-93-5p was associated with attenuation of MI-induced cardiomyocyte damage and abrogated the detrimental effect of circHIPK3. CircHIPK3 acted as a sponge for miR-93-5p, thereby enhancing the activation of the Rac1/PI3K/AKT signaling pathway. In conclusion, circHIPK3 suppression was associated with overexpression of miR-93-5p and inhibition of the Rac1/PI3K/Akt signaling pathway, leading to amelioration of MI-induced cardiac dysfunction (195).

The novel circRNA, circMAT2B was shown to play a major role in moderating glucose metabolism in hypoxic conditions (196). Moreover, elevated levels of circMAT2B were found in hepatocellular carcinoma patients, suggesting it could be a target for hepatocellular carcinoma treatment. Nevertheless, its role in MI is still unclear.

Zhu et al. investigated how circMAT2B affects MI. An oxygen-glucose deprivation (OGD)-induced H9c2 cell model was used to simulate MI. H9c2 cells were transfected with ex-circMAT2B plasmid for overexpression, si-circMAT2B for a knockdown, a miR-133 inhibitor, and appropriate controls. The expression levels of miR-133 and circMAT2B were evaluated using qRT-PCR. Apoptosis, reactive oxygen species (ROS) production, cell viability, and secretion of inflammatory cytokines were evaluated using flow cytometry, ROS assay kit, CCK-8 assay, and ELISA, respectively. Furthermore, Western blotting was employed to identify apoptosis and related pathways. It was found that OGD treatment significantly increased circMAT2B expression. Additionally, circMAT2B knockdown dramatically reduced the OGD-stimulated increase in apoptosis, ROS production, and the expression of inflammatory cytokines. SI-circMAT2B increased miR-133 expression. CircMAT2B suppression abrogated OGD-induced H9c2 cell damage and relieved the OGD-stimulated inhibition of the Raf/MEK/ERK and PI3K/AKT signaling pathways *via* upregulation of miR-133. Collectively, circMAT2B suppression could inhibit inflammation in OGD-induced cardiomyocyte damage in H9c2 cells by upregulating miR-133 (197).

The circRNA Postn (circPostn) is thought to affect cancer development *via* modulating cell apoptosis and proliferation (198). One *in vivo* study showed that circPostn is up-regulated in MI (199). Nonetheless, its role in MI-induced myocardial damage and regeneration is not yet clear.

Cheng et al. investigated the effects of circPostn on myocardial damage and remodeling following MI. They found higher plasma levels of circPostn in patients suffering from MI, as well as mice and human cardiomyocytes treated with H/R. CircPostn knockdown considerably reduced MI-induced myocardial damage and infarct size. CircPostn knockdown also increased the left ventricular FS and EF, as well as decreased the left ventricular anterior wall thickness in diastole (LVAWd) and the left ventricular posterior wall thickness in diastole (LVPWd). The expression of collagen 1α1 and collagen 3α1 was increased in MI *in vivo* but was reduced when circPostn was depleted. Collagen and smooth muscle actin protein expression was increased in MI *in vivo* and decreased by circPostn knockdown. Moreover, suppression of circPostn decreased the expression of the atrial natriuretic peptide as well as brain natriuretic peptide. Also, the circPostn depletion was able to decrease cardiomyocyte apoptosis *in vivo*. CircPostn acted mechanically as a miR-96-5p sponge and miR-96-5p targeted BNIP3 in human cardiomyocytes, where circPostn increased BNIP3 level through miR-96-5p targeting. circPostn enhanced H/R-stimulated cardiomyocyte damage *via* affecting the miR-96-5p/BNIP3 axis. Therefore, the authors infer that circPostn results in MI-stimulated myocardial damage and heart remodeling *via* moderating the miR-96-5p/BNIP3 axis. The results illustrate a unique understanding of the mechanism behind the regulatory effects of circPostn on MI-stimulated heart dysfunction. miR-96-5p, circPostn, and BNIP3 can be considered as possible therapeutic for the management of MI-stimulated heart damage (200) (Table 3).

**Table 3:** Reports of changes in circRNA expression in laboratory models and patients with MI.

**Circular RNA**	**Expression**	**Target**	**Mechanism**	**Model (*In vitro*, *in vivo*, human)**	**Tissue, blood, cell**	**Reference**
Circ-MACF1	Down	miR-500b-5p	Inhibited apoptosis, improved myocardial function	*In vivo, in vitro*	Tissue, cardiomyocytes	(201)
Circ-SNRK	Down	miR-103-3p	Increased proliferation, reduced apoptosis	*In vivo, in vitro*	Tissue, cardiomyocytes	(202)
Circ-Amotl1	Down	PDK1, AKT1	Reduced apoptosis and promoted cardiac repair	*In vivo, in vitro*, human	Tissue	(203)
Circ-0000064	Down	–	Attenuated autophagy	*In vivo*	Tissue	(204)
Circ-ACR	Down	Pink1	Repressed autophagy, decreased myocardial infarct sizes	*In vitro, in vivo*	Tissue, cardiomyocytes	(100)
Circ-CDYL	Down	miR-4793-5p	Promoted cell proliferation, improved heart function	*In vivo, in vitro*	Tissue	(205)
Circ-Fndc3b	Down	FUS	Reduced apoptosis, enhanced neovascularization	*In vivo, in vitro*, human	Tissues, cardiomyocytes	(199)
Circ-RCAN2	Down	–	–	*In vivo, in vitro*	Tissue	(206)
Circ-MICRA	Down	–	–	Human	Blood	(207)
Circ-LAS1L	Down	miR-125b	Reduced cell proliferation and migration, induced apoptosis	*In vitro*, human	Blood, cardiac fibroblasts	(208)
Circ-NFIB	Down	miR-433	Decreased cell proliferation, reduced cardiac fibrosis	*In vivo, in vitro*	Tissue, cardiac fibroblasts	(209)
Circ-PAN3	Down	miR-421	Inhibited autophagy, reduced apoptosis	*In vivo, in vitro*	Tissue, HCMs	(210)
Circ-UBXN7	Down	miR-622	Decreased apoptosis and inflammation	*In vitro, in vivo*,	Tissue, H9c2 cells	(211)
Circ-C12orf29	Down	–	–	*In vitro, in vivo*	Tissue	(206)
Circ-JARID2	Up	miR-9-5p	Inhibited cell viability, promoted apoptosis and inflammatory response	*In vitro*	H9c2 cells	(212)
Circ_0124644	Up	miR-590-3p	Promoted cardiomyocytes injury via regulating SOX4	*In vitro*, human	Blood, AC16 cells	(213)
Circ-ROBO2	Up	miR-1184	Induced apoptosis	*In vivo, in vitro*	Tissue	(192)
Circ-HIPK3	Up	miR-93-5p	Inhibited cardiomyocyte proliferation and induced apoptosis	*In vivo, in vitro*	Tissue, HL-1 cells	(195)
Circ-Ttc3	Up	miR-15b	Decreased ATP depletion and apoptosis	*In vivo, in vitro*	Tissue, cardiomyocytes	(214)
Circ-Arhgap12	Up	miR-135a-5p	Enhanced apoptosis and oxidative stress	*In vivo*	Tissue	(215)
Circ-ZNF292	Up	BNIP3	Increased cell viability, decreased apoptosis and autophagy	*In vitro*	H9c2 cells	(216)
Circ-MAT2B	Up	miR-133	Induced inflammation	*In vitro*	H9c2 cells	(197)
Circ-Helz	Up	miR-133a-3p	Induced inflammation and pyroptosis, increased myocardial infarct size, decreased cardiac function	*In vivo, In vitro*	Tissues, cardiomyocytes	(217)
Circ-SLC8A1	Up	miR-133a	-	*In vivo, in vitro*, human	Tissue, cardiocytes	(218)
Circ-FASTKD1	Up	miR-106a	Reduced the viability, migration, and angiogenesis	*In vitro, in vivo*	HUVECs, HCMECs	(219)
Circ-PAN3	Up	miR-221	Increased cell proliferation and migration, induced cardiac fibrosis	*In vivo, in vitro*	Tissue, cardiac fibroblasts	(220)
Circ-000203	Up	miR-26b-5p	Increased expressions of pro-fibrotic genes	*In vitro, in vivo*	Tissue	(221)
Circ-Ube3a	Up	miR-138-5p	Promoted cell proliferation and migration, aggravated myocardial fibrosis	*In vivo, in vitro*	M0M-SEVs, M2M-SEVs	(222)
Circ-001654, circ-091761, circ-405624, circ-406698	Up	miR-491-3p, miR-646, miR-603, miR-922	–	Human	Blood	(223)
Circ-0023461	Up	miR-370-3p	Decreased cell viability, proliferation and migration, increased apoptosis, oxidative stress, and inflammation	*In vitro*, human	Blood, AC16 cell line	(224)

## Exosomal non-coding RNAs and myocardial infarction

Exosomes are bilayer-surrounded membrane vesicles measuring from 30 to 100 nm in diameter and are secreted by most cell types. Exosomes can act as carriers to transport biomolecules including, DNA, proteins, RNA, and lipids (225, 226).

Exosomes can display cell molecules characteristic of their source cells on their surface and when they bind to recipient cells, they release their contents into the cytosol of the recipient cells through receptor-ligand interactions, endocytosis, and fusion with the cell membrane, thereby modifying the recipient cell functions (227). A relatively large number of proteins, miRNAs, and lncRNAs can be detected inside exosomes. Emerging evidence suggests that exosomes can act as regulators of cell differentiation, proliferation, and apoptosis in CVDs (228–230). By acting as ceRNA, they can bind to specific miRNAs and act as miRNA sponges in cells. Consequently, they can reduce the activity of miRNAs and thus regulate the expression of their target genes (231).

Exosomal ncRNAs act as regulators of myocardial structure and function in CADs, thus providing new insight into the mechanisms and therapeutic targets for the diagnosis and treatment of these diseases (232). Sirt1 is involved in immune responses and is known as a regulator of inflammation in CVDs (233, 234). It plays a critical role in the regulation of cardiac cell development and CVDs (235, 236). In MI patients, Sirt1 is downregulated, whereas its upregulation could alleviate MI-induced myocardial damage (237). Sirt1 activation could inhibit NLRP3 inflammasome activation and subsequent caspase-1 cleavage and IL-1β secretion, suggesting the protective effect of Sirt1 on vascular endothelial cells (238).

Mao et al. investigated the effects and the underlying mechanism of lncRNA KLF3-AS1 contained in exosomes derived from human mesenchymal stem cells (hMSCs), on the pyroptosis of cardiomyocytes and the treatment of MI (239). Exosomes were transfected with KLF3-AS1 and tested *in vitro* and *in vivo*. The effects of exosomal KLF3-AS1 on cell viability, MI area, pyroptosis, and apoptosis were assessed. The dual-luciferase reporter assay was used to determine correlations between KLF3-AS1, miR-138-5p, and Sirt1. Transfection of miR-138-5p and sh-Sirt1 into normal cardiomyocytes was performed to see whether an increase in miR-138-5p or sh-Sirt1 could affect the cardioprotective activity of KLF3-AS1. The delivery of KLF3-AS1 in exosomes resulted in reduced infarct size, less apoptosis, and pyroptosis, and reduced MI progression. KLF3-AS1 can bind to miR-138-5p to modulate the expression of Sirt1. It was found that inhibition of miR-138-5p was associated with decreased pyroptosis and ameliorated the effects of MI. The authors concluded that lncRNA KLF3-AS1 in exosomes released from hMSCs could bind to miR-138-5p to moderate Sirt1 expression to suppress pyroptosis and slow MI progression (239).

Recently, Chen et al. conducted an *in vitro* study to evaluate the role of exosome-mediated lncRNAs ZEB1-AS1 (Zinc finger e-box-binding homeobox 1 antisense 1) and its underlying mechanisms in atherosclerosis (240). Exosomes were extracted from oxidized low-density lipoprotein (ox-LDL)-treated human umbilical vein endothelial cells (HUVECs). They demonstrated that exo-lncRNA ZEB1-AS1 derived from ox-LDL-induced HUVECs amplified cell injuries by miR-590-5p/ ETS1 (E26 oncogene homolog 1) axis through the TGF-β/Smad pathway, suggesting that stopping ZEB1-AS1 might be an efficient strategy to treat atherosclerosis (240).

It has been shown that serum MMP-9 could be used to differentiate between MI and unstable angina (UA) with a sensitivity and specificity of 80%. MMP-9 levels also predicted unfavorable outcomes in ST-elevation MI (STEMI) patients with a sensitivity of 72.4% and a specificity of 83% (241). Because MI patients show an imbalance between MMPs and their inhibitors (TIMPs), an increase in MMP leading to the degradation of the fibrous cap can be a major cause of plaque instability (242, 243). Increased MMP-9 levels stimulate plaque rupture and result in acute MI. Moreover, high levels of inflammatory cytokines following acute MI promote the additional production and release of MMP-9 (244).

Exosomal miR-221/222 secreted by human aortic smooth muscle cells (HAOSMCs) was found to inhibit HUVECs autophagy in by partially modulating the PTEN/Akt signaling pathway when HUVECs and HAOSMCs were co-cultured (245). According to research on the influence of exosomes on cardiac fibroblasts, hypoxia upregulates the lncRNA AK139128 expression in cardiomyocytes and exosomes, and exo-lncRNA AK139128 derived from hypoxic cardiomyocytes endorses apoptosis, prevents cell proliferation, and modulates fibroblast activity (246). One study showed that the increased expression of miR-125b of mesenchymal stem cells-secreted exosomes enhances myocardial cell survival in rats after I/R by regulating Sirt7, decreasing myocardial cell apoptosis and inflammatory response, and increasing heart function (247). Another investigation found that miR-425 and miR-744 levels in plasma exosome samples from heart failure patients were considerably lower (248). Overexpression of miR-425 or miR-744 in cultured cardiac fibroblasts suppresses TGF1 expression and dramatically decreases angiotensin-induced collagen production and fibrogenesis (248, 249).

Numerous studies have shown that secreted exosomes from myocardial cells contain some microRNA such as miR-125b, miR-126, miR-25-3p, miR-144, and miR-146a which are overexpressed and exert an anti-atherosclerotic role by stopping myocardial apoptosis and facilitating ischemic cardiac repair (250–254). In addition, exosomal miRNAs, miR-25-3p and miR-146a also inhibit the inflammatory response, while exosomal miR-301 prevents myocardial autophagy (254–256). Besides, according to Wang et al., enhanced expression of EXO-MSC-derived miR-21 plays a cardioprotective role by inhibiting apoptosis, promoting angiogenesis, and increasing cell survival through the PTEN/Akt pathway (257).

Chen et al. investigated the association between acute MI and plasma exosomes containing lncRNA NEAT1 (258). They categorized the participants into three groups: the control group, the unstable angina group, and the acute STEMI, overexpression of NEAT1 and MMP-9 was found and they were positively correlated with each other. Furthermore, lower levels of miR-204 were found in STEMI patients and no significant correlation was found between expression levels of NEAT1 or MMP-9 and miR-204. The authors concluded that miR-204, exosomal NEAT1, and MMP-9 could all be considered diagnostic markers for STEMI (258) (Table 4).

**Table 4:** Reports of changes in exosomal ncRNAs in laboratory models and patients with MI.

**Exosomal cargo**	**Expression**	**Target**	**Source**	**Mechanism**	**Model (*In vitro*, *in vivo*, human)**	**Reference**
miR-1,915-3p, miR-6,741-5p, miR-6,850-5p, miR-7,108-5p, miR-6,803-5p	Down	–	Serum-derived	–	Human	(259)
miR-6,798-3p, miR-4,486, miR-7,975, miR-4,634, miR-3,195, miR-1,227-5p	Down	–	Serum-derived	–	Human	(259)
miR-3,656	Down	CSK	Serum-derived	–	Human	(259)
miR-4,507	Down	PEBP1	Serum-derived	–	Human	(259)
lncRNA SOCS2-AS1	Down		Plasma	–	Human	(260)
miR-183	Up	PPP2CB, PPP2CA, PRKCA, PPP2R5C, PPP2R2A	Plasma-derived	Involved in cell communication, protein kinase activity regulation and adrenergic signaling in cardiomyocytes	Human	(261)
lncRNA-MALAT1	Up	miR-92a	Cardiac myocyte-derived	Improved angiogenesis, decreased infarct size	*In vivo, in vitro*	(262)
lncRNA-H19	Up	miR-675	Mesenchymal stem cells-derived	Promoted angiogenesis	*In vivo, in vitro*	(263)
lncRNA-KLF3-AS1	Up	miR-138-5p	Mesenchymal stem cells-derived	Decreased cell apoptosis and pyroptosis, and attenuated MI progression	*In vivo, in vitro*	(239)
lncRNA-UCA1	Up	miR-873-5p	Mesenchymal stem cells-derived	Decreased apoptosis	*In vitro, in vivo*, human	(264)
lncRNA-NEAT1	Up	miR-204	Serum	–	Human	(258)
lncRNAs ENST00000556899.1, ENST00000575985.1	Up	–	Plasma	–	Human	(265)
circ-0001273	Up	–	Umbilical cord mesenchymal stem cells-derived	Inhibited apoptosis, promoted MI repair	*In vivo, in vitro*	(266)
lncRNA RNCR3	Up	miR-185-5p	Endothelial cell, and vascular smooth muscle cell-derived	Decreased inflammatory factor releases, induced proliferation and migration, reduced apoptosis	*In vitro, in vivo*	(267)
lncRNA MALAT1	Up	miR-92a	Cardiac myocyte-derived	Enhanced neovascularization	*In vivo, in vitro*	(262)
lncRNA-NEAT1	Up	miR-142-3p	Mesenchymal stem cell-derived	Inhibited apoptosis	*In vitro*	(268)
miR-125b-5p	Up	–	Mesenchymal stem cell-derived	Inhibited apoptosis	*In vivo, in vitro*	(250)
miR-106a-3p	Up	VSMCs	Ox-LDL	Promoted cell proliferation, repressed apoptosis	*In vitro*	(269)
miR-221-3p	Up	PTEN	Senescent Mesenchymal Stem Cells-derived	Improved angiogenesis, migration and proliferation, suppressed apoptosis	*In vitro, in vivo*	(270)
BMSC-Exo	Up	miR-486-5p	Bone-marrow stromal cells-derived	Induced proliferation, reduced apoptosis	*In vitro, in vivo*	(271)
lncRNA AK139128	Up	Fibroblasts	Cardiomyocytes-derived	Promoted apoptosis, inhibited proliferation, migration, and invasion in Cardiac Fibroblasts	*In vitro, in vivo*	(246)
miR-221/222	Up	PTEN	Human aortic smooth muscle cell-derived	Inhibited autophagy in human umbilical vein endothelial cells	*In vitro*	(245)
lncRNA HIF1a-AS1	Up	–	Plasma	–	Human	(272)
lncRNA UCA1	Up	miR-873	Mesenchymal stem cells-derived	Decreased apoptosis	*In vivo*, human	(264)

## Conclusion and future perspectives

We comprehensively review the mechanisms of epigenetics such as DNA methylation, histone modification, and non-coding RNA in various cardiovascular diseases. Moreover, basic and clinical studies on epigenetic therapy for these diseases are summarized.

Existing treatments for CADs have not been equally effective in all patients, suggesting that interindividual variability is an aspect that plays a significant role in personalized treatment. Genetic testing and genomic mapping combined with emerging transcriptional technologies such as microarrays and RNA-Seq, as well as methylation or acetylation patterns and bioinformatics tools can help determine the genetic and epigenetic structures that define an individual's risk profile and identify novel therapeutic epigenetic targets. In this context, the development of small molecule drugs as epigenetic or miRNA modulators (e.g., HDAC inhibitors) has opened up new approaches for the treatment of cardiovascular diseases. Evidence has revealed that ncRNAs can act either as pro-MI or anti-MI molecules by modulating signaling pathways relevant to myocardial cell death or cardiomyocyte regeneration. Up to now, many advances have identified ncRNA-mediated signaling pathways involved in MI, which have contributed to improvement in our understanding of cardiovascular pathogenesis. Abnormal plasma levels of many ncRNAs have been observed in patients with MI, which suggests their potential in the diagnosis and treatment of MI. Importantly, some ncRNA-based therapeutic approaches have shown promising results in animal models of MI, which provides some hope that this approach could be used for the clinical management of MI soon.

The role of exosomal ncRNAs in the progression and development of CADs, such as atherosclerosis, acute coronary syndrome, HF, myocardial I/R injury, and pulmonary hypertension has been the focus of many recent studies. Tissue-specific changes of exosomal ncRNAs could play a role in the initiation and progression of these complications. In terms of biomarkers, exosomal ncRNAs could be superior compared to non-exosomal ncRNAs. Many ncRNAs are harbored inside exosomes, which protects them from degradation and enhances their stability. Moreover, exosomal ncRNAs exist in considerable amounts in different body fluids that can be non-invasively sampled. Hence, exosome ncRNAs are expected to become a new tool for the diagnosis and treatment of CADs.

Considering the epigenetic roles of some RNAs and their capacities to control gene expression, the establishment of novel approaches for modulating the expression of some ncRNAs supported by the development of epigenetic-related drugs with higher specificity, fewer side effects, and lower drug resistance for various types of CADs will be the goal of future development. It is hoped that in the near future, the discovery and continuous research and development of epigenetic regulatory drugs for CADs will have a wider application perspective and will be more beneficial for patients with cardiovascular diseases. To this end, more large-scale basic and clinical trials are still needed to better understand the epigenetic molecular mechanism regulating CADs and to find more strategies to avoid and treat cardiovascular diseases, so as to better guide clinical treatments.

## Author contributions

HM was involved in conception, design, statistical analysis, and drafting of the manuscript. SF, AM, MP, MS, MZ, FZ, ST, MR, AS, and MH contributed to data collection and manuscript drafting. All authors approved the final version for submission.

## Conflict of interest

The authors declare that the research was conducted in the absence of any commercial or financial relationships that could be construed as a potential conflict of interest.

## Publisher's note

All claims expressed in this article are solely those of the authors and do not necessarily represent those of their affiliated organizations, or those of the publisher, the editors and the reviewers. Any product that may be evaluated in this article, or claim that may be made by its manufacturer, is not guaranteed or endorsed by the publisher.
